# RETRACTED: Kulanayake et al. Role of Protein VII in the Production of Infectious Bovine Adenovirus-3 Virion. *Viruses* 2024, *16*, 1323

**DOI:** 10.3390/v17081133

**Published:** 2025-08-19

**Authors:** Shermila Kulanayake, Barinder Singh, Faryal Dar, Suresh K. Tikoo

**Affiliations:** 1 Vaccinology & Immunotherapeutics Program, School of Public Health, University of Saskatchewan, Saskatoon, SK S7N 5E5, Canada; shermila.kulanayake@usask.ca (S.K.); uyl039@mail.usask.ca (F.D.); 2 Vaccine and Infectious Disease Organization (VIDO), University of Saskatchewan, Saskatoon, SK S7N 5E5, Canada; qal946@mail.usask.ca; 3 Veterinary Microbiology, Western College of Veterinary Medicine, University of Saskatchewan, Saskatoon, SK S7N 5B4, Canada

The journal retracts the article “Role of Protein VII in the Production of Infectious Bovine Adenovirus-3 Virion” [1], cited above.

Following publication, concerns were brought to the attention of the publisher regarding the integrity of a number of images presented in this publication [1].

Adhering to our complaints procedure, an investigation was conducted by the Editorial Office and the Editorial Board confirmed image issues in Figures 1b, 2d (pUC304a,9dpi), 6b (IVa2), 7a (Tubulin), and 8 (CsCl). While the authors cooperated with the Editorial Office during the investigation and provided supporting material, they were unable to satisfactorily explain the image irregularities. As a result, the Editorial Board has lost confidence in the reliability of the findings and decided to retract this article [1] as per MDPI’s retraction policy (MDPI|Research and Publication Ethics https://www.mdpi.com/ethics#_bookmark30).

This retraction was approved by the Editor-in-Chief of the journal Viruses.

The authors agreed to this retraction.
